# Impact of Long-Rope Jumping on Monoamine and Attention in Young Adults

**DOI:** 10.3390/brainsci11101347

**Published:** 2021-10-13

**Authors:** Masatoshi Yamashita, Takanobu Yamamoto

**Affiliations:** 1 Graduate School of Advanced Integrated Studies in Human Survivability, Kyoto University, Kyoto 606-8306, Japan; 2 Department of Psychology, Tezukayama University, Nara 631-8585, Japan; takaoxford@gmail.com

**Keywords:** long-rope jumping, attention, 3-methoxy-4-hydroxyphenylglycol, 5-hydroxyindoleacetic acid

## Abstract

Previous research has shown that rope jumping improves physical health; however, little is known about its impact on brain-derived monoamine neurotransmitters associated with cognitive regulation. To address these gaps in the literature, the present study compared outcomes between 15 healthy participants (mean age, 23.1 years) after a long-rope jumping exercise and a control condition. Long-rope jumping also requires co-operation between people, attention, spatial cognition, and rhythm sensation. Psychological questionnaires were administered to both conditions, and Stroop task performance and monoamine metabolite levels in the saliva and urine were evaluated. Participants performing the exercise exhibited lower anxiety levels than those in the control condition. Saliva analyses showed higher 3-methoxy-4-hydroxyphenylglycol (a norepinephrine metabolite) levels, and urine analyses revealed higher 3-methoxy-4-hydroxyphenylglycol and 5-hydroxyindoleacetic acid (a serotonin metabolite) levels in the exercise condition than in the control. Importantly, urinary 5-hydroxyindoleacetic acid level correlated with salivary and urinary 3-methoxy-4-hydroxyphenylglycol levels in the exercise condition. Furthermore, cognitive results revealed higher Stroop performance in the exercise condition than in the control condition; this performance correlated with salivary 3-methoxy-4-hydroxyphenylglycol levels. These results indicate an association between increased 3-methoxy-4-hydroxyphenylglycol and attention in long-rope jumping. We suggest that long-rope jumping predicts central norepinephrinergic activation and related attention maintenance.

## 1. Introduction

Exercise has beneficial effects on psychophysiological well-being and brain function [1,2]. However, a monotonous or complicated exercise training program may not be the most enjoyable or easily performed exercise. In a society suffering from increasing health issues, identifying simple and enjoyable exercises that can effectively mitigate mental illness-related cognitive and brain dysfunction is important.

Being a simple and aerobic rhythmic exercise, the rope jumping program was introduced as a teaching tool for improving physical health in Japanese elementary schools in the early 1900s. Rope jumping co-ordinates movements of the upper and lower body to maintain balance and rhythm. Pitreli and O’Shea reported that rope jumping combines the angular momentum of the rope and vertical displacement of the body and involves upper and lower synchrony where positioning and timing are critical [3]. Given that time perception is involved in the processing of timing information [4], the rope jumping skill may be associated with superior cognitive functions, including spatial-temporal perception. A previous study reported that young adults who engaged in rope jumping had higher levels of selective attention than young adults who did not exercise [5]. Rope jumping improves C-reactive protein levels and bone density in adolescents [6,7]. However, little is known about how rope jumping affects monoaminergic activity related to cognitive performance.

The monoamine neurotransmitters norepinephrine, serotonin, and dopamine have been implicated in various cognitive functions [8,9]. Norepinephrine is involved in the regulation of the dorsal and ventral attention networks and thereby in reorienting and switching attention [10,11]. Additionally, some positron emission tomography studies have reported associations between dopamine or serotonin availability and attention in healthy individuals [12,13]. These findings indicate that monoamines are critically linked to selective attention. However, investigations of monoamines have been limited to neuroimaging, and little is known about the association between direct monoamine levels and cognitive function after rope jumping. Does rope jumping, which enhances attention in young adults, change monoamine levels in the central nervous system (CNS)?

Biochemical studies have shown an association between monoamine levels in the peripheral nervous system (PNS) and the CNS [14,15]. Although 3-methoxy-4-hydroxyphenylglycol (MHPG; a norepinephrine metabolite) levels in the plasma and cerebrospinal fluid (CSF) were related to the central norepinephrinergic system according to some studies [16,17], several other studies failed to demonstrate this association [18,19]. This discrepancy may be associated with the stress of invasive blood and/or CSF extraction. In contrast, salivary MHPG (sMHPG), delivered via blood circulation, is a non-invasive marker for detecting changes in the central norepinephrinergic system, as sMHPG has been reported to correlate with plasma and CSF MHPG levels [20,21]. Additionally, a growing body of evidence indicates that sMHPG levels can predict cognitive performance and mental health associated with central norepinephrinergic activity [22,23]. Moreover, it has been reported that urinary MHPG (uMHPG), which does not require invasive sampling, originates in the CNS [24,25]. Urinary homovanillic acid (uHVA; a dopamine metabolite) and 5-hydroxyindoleacetic acid (u5-HIAA; a serotonin metabolite) are transported from the brain via the organic anion transporter 3 system (OAT 3) in the blood-brain barrier [26,27]. Moreover, the reduction of brain serotonin levels was correlated with lower u5-HIAA levels [14]. Such monoamine metabolites in the saliva and urine can serve as non-invasive and useful markers for detecting central monoamine activity in humans.

The effect of rope jumping exercise on cognitive regulation-relevant monoamine neurotransmitters has received scant attention, and little is known about monoamine metabolite levels in the saliva and urine after performing rope jumping exercise. However, given the effect of rope jumping exercise on attention performance and physiological function, it is important to clarify whether rope jumping exercise also affects the association between monoamines and attention. To determine this association, we used long-rope jumping as an experimental condition, as it is a rhythmic exercise requiring considerable co-operation, attention, spatial cognition, and rhythm sensation when performed with multiple persons.

The present study addresses two research questions. First, does the rhythmic exercise of long-rope jumping enhance monoamine metabolite levels? Second, if improvement is observed with the rhythmic exercise, does it lead to a corresponding improvement in attention performance as measured by the Stroop task? To address these questions, we compared monoamine metabolites levels in saliva and urine and Stroop performance between long-rope jumping and non-exercise conditions. Based on the monoamine metabolite differences between conditions, we further examined the associations between monoamine metabolite levels and Stroop performance.

## 2. Materials and Methods

### 2.1. Participants

The Psychological Research Ethics Committee of Tezukayama University approved the protocol (approval number 27−15), and all study participants provided informed consent. Twenty-four university students (10 women and 14 men, aged 20–33 years) participated in this study; they were recruited from Tezukayama University. Individuals with a history of neurological, cardiovascular, or psychiatric illness, smoking, or use of drugs (e.g., tranquilizers or hypnotics) were excluded. Since previous studies have reported associations between menstruation and monoamine metabolism and physical activity-related physiological systems [28,29,30], five women were also excluded from the analysis because of ongoing menstruation and/or physical deconditioning. Four men were excluded because of physical deconditioning. The final analysis included 15 healthy students (5 women and 10 men; mean age = 23.1 years, *SD* = 3.4; mean education = 16.6 years, *SD* = 2.2).

### 2.2. Experimental Conditions

**Figure 1:**
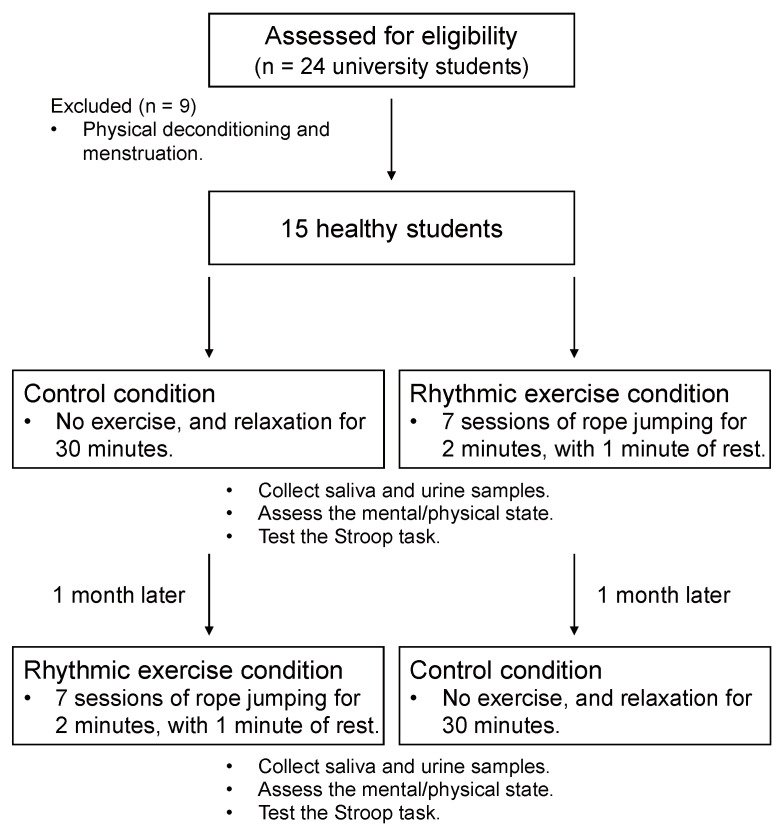
Study flow diagram.

We selected long-rope jumping as the rhythmic exercise condition (EC). The rhythmic exercise performed by four persons per group consisted of seven sessions of jumping for 2 min, with 1 min of rest after each session. The rope made one rotation per second, and a metronome was used to measure this rate. Moreover, the mean intensity in long-rope jumping was 56.7% (mean heart rate during rope-jumping = 109.3 bpm, *SE* = 4.5; mean maximum heart rate = 192.7 bpm, *SE* = 0.7), indicating mild rhythmic exercise for maintaining health. The subjects had free access to water during the rhythmic exercise. In contrast, the control condition (CC) was set as a period of relaxation in a private room for 30 min. During the relaxation period, the participants were allowed to drink only water, and were not allowed to use the phone, read, exercise, or sleep. The present study was a within-subjects counterbalanced design (Figure 1). First, all participants were randomly assigned to either of the two conditions (control or exercise). After experiencing either of the two conditions, the participants were treated with the unexperienced condition 1 month later.

### 2.3. Determination of MHPG in Saliva

Saliva was collected by placing two swabs on the sublingual gland in both conditions. Saliva was immediately transferred to a polypropylene tube with 2.5% perchloric acid. The tube was centrifuged at 10,000× *g* for 10 min at 4 °C. The obtained supernatant was stored at −78 °C until assayed by high performance liquid chromatography (HPLC; Nanospace SI-2 3001, Shiseido, Tokyo, Japan) equipped with an electrochemical detector (Nanospace SI-2 3005, Shiseido, Japan). The supernatant was directly injected into the HPLC system. sMHPG concentration was measured using HPLC with an electrochemical detector (voltage: 700 mV) and a chromate recorder (C-R8A, Shimadzu Co, Kyoto, Japan). The mobile phase consisted of 15% methanol in a solution (pH 4.13) containing 30 mM citric acid, 10 mM disodium hydrogen phosphate, 0.5 mM sodium octyl sulphate, 50 mM sodium chloride, and 0.05 mM ethylenediaminetetraacetic acid. This was pumped through a 5-μM C_18_ column (150 mm × 4.6 mm; TSK gel, ODS-80TM, Tosoh, Tokyo, Japan) at a flow rate of 0.7 mL/min. sMHPG retention time was approximately 6 min. However, s5-HIAA and sHVA were not detected in either condition.

### 2.4. Determination of Monoamine Metabolites in Urine

The collected urine in both conditions was diluted with 6.7 mM hydrochloric acid and 2.5% perchloric acid. The mixture was centrifuged at 10,000× *g* for 10 min at 4 °C to separate albumin. The obtained supernatant was stored at −78 °C until the HPLC assay. The mobile phase, column, flow rate, and detection system were the same as those used for sMHPG determination. The retention times of uMHPG, u5-HIAA, and uHVA were 6, 12, and 18 min, respectively.

### 2.5. Psychological Measurements

The profile of mood states (POMS) is a 30-item questionnaire used to measures six basic mood states on a 5-point Likert scale (0 = not at all to 4 = very): tension (e.g., “I feel fidgety”), depression (e.g., “I feel dark”), anger (e.g., “I feel intense anger”), vigor (e.g., “I have vigour”), fatigability (e.g., “I was tired”), and confusion (e.g., “I have not gathered my thoughts”). In addition, the social behavioral questionnaire included 17 question items that measure two basic social dimensions on a 5-point Likert scale (0 = not at all to 4 = very): individual (e.g., “I value my own individuality”) and social (e.g., “I stay coordinated with other people”) orientations.

### 2.6. Cognitive Measurements

Selective attention and cognitive flexibility were measured to compare the rhythmic EC and CC. The cognitive test consisted of the classical version of the Stroop task as previously reported [31]. Subjects were asked to name the ink color in congruent words (e.g., the word red written in red ink) and incongruent words (e.g., the word blue written in green ink, or the word yellow written in red ink). Under both congruent and incongruent conditions, the words were printed on an A3 size paper with a font size of 16 for each word. The participants were asked to answer with the word or color on the paper as quickly and accurately as possible for 60 seconds/task, and the number of correct responses was recorded. To offset the effect of task order, the order of tasks was counterbalanced for all participants.

### 2.7. Procedure

The participants were instructed to refrain from consuming alcohol, coffee, fish, red beef, and blue cheese for at least 24 h before the experiment. In addition, they were asked to fast with water during the 90-min period before saliva and urine collection. On the day of the experiment, participants urinated, brushed their teeth with water, and then rested in a private room for 30 min. In the CC, saliva and urine were collected 30 min after the relaxation period. Next, the mental/physical state of the participants was assessed using questionnaires, and their cognitive function was tested using the Stroop task. In the EC, saliva and urine were collected 30 min after completing the rhythmic exercise. Next, questionnaires were administered to determine the mental and physical state of the participants, and the Stroop task was administered to determine their cognitive function.

### 2.8. Statistical Analyses

Psychological, biochemical, and cognitive data were compared between the rhythmic EC and CC using the paired sample *t*-test in IBM-SPSS, version 25 (IBM Corp., Armonk, NY, USA). However, multiple tests may induce a type I error for overestimating significant effects under no-correction, or a type II error for underestimating significant effects under conservative correction such as the Bonferroni methods. The resampling method (e.g., permutation) can be used to estimate adjusted *p*-values while avoiding parametric assumptions about the joint distribution of the test statistics [32,33]. We conducted a permutation test using MATLAB R2020a (The Mathworks Inc., Natick, MA, USA) for the validation of the original exercise effects detected under the uncorrected α-level threshold. For each experimental data, all samples were randomized together and resampled to obtain a dummy *t*-value. This procedure was repeated 10,000 times for each of the 8 psychological, 4 biochemical, and 2 cognitive data. We pooled a total of *t*-values (80,000 *t*-values: 10,000 resampling × 8 psychological data, 40,000 *t*-values: 10,000 resampling × 4 biochemical data, 20,000 *t*-values: 10,000 resampling × 2 cognitive data) and created a unique permutation *t*-distribution to obtain a single adjusted α-level threshold (the top five percentile ranks in the distribution) of each *t*-value in the psychological, biochemical, and cognitive data.

Finally, correlations were calculated using the Pearson correlation coefficient in IBM-SPSS. For these multiple coefficients, validation tests for correlations were performed in a permutation test using MATLAB R2020a. To examine the correlation in a given pair of variables (e.g., urinary 5-HIAA and salivary MHPG), a dummy coefficient was obtained by correlating the two variables randomly across participants. This procedure was repeated 10,000 times for each of the 16 correlations. We pooled a total of 160,000 dummy coefficients (10,000 resampling × 16 correlations) and created a unique permutation coefficient distribution to obtain a single adjusted α-level threshold (the top five percentile ranks in the distribution). For all analyses, *p* < 0.05 was considered statistically significant.

## 3. Results

### 3.1. Psychological Scores

The psychological data are shown in Table 1. There was a significant difference between the two conditions in the POMS anxiety score (*t*_(14)_ = 5.28, *p* < 0.001, *d* = 0.96). The *t*-values in the POMS anxiety score were higher than the adjusted significance level threshold (*t*_(14)_ = 3.24) obtained in the permutation test. In contrast, there were no significant differences in POMS depression, anger, vigor, fatigability and confusion scores, and individual and social orientation between the two conditions. These results indicate that, compared with the CC, the rhythmic exercise of long-rope jumping reduced anxiety levels.

### 3.2. Rope Jumping Exercise-Related Monoamine Metabolite Changes and Cognitive Effects

**Figure 2:**
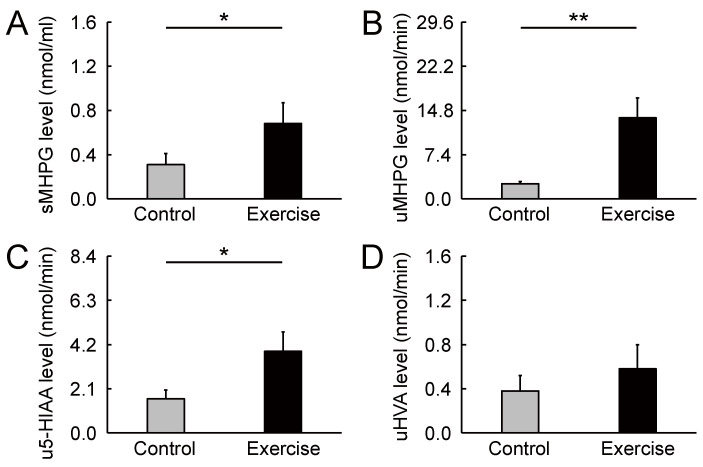
Change in monoamine metabolite levels stimulated by rhythmic rope jumping exercise. (**A**) Compared with the control condition, rhythmic exercise increased salivary 3-methoxy-4-hydroxyphenylglycol levels 30 min after exercise. (**B**,**C**) rhythmic exercise participants showed increased urinary 3-methoxy-4-hydroxyphenylglycol and 5-hydroxyindoleacetic acid levels when compared with participants in the control condition. (**D**) There was no significant difference between conditions in urinary homovanillic acid level. Parameters are indicated as mean (*SE*). * *p* < 0.05, ** *p* < 0.01. sMHPG, salivary 3-methoxy-4-hydroxyphenylglycol; uMHPG, urinary 3-methoxy-4-hydroxyphenylglycol; u5-HIAA, urinary 5-hydroxyindoleacetic acid; uHVA, urinary homovanillic acid; *SE*, standard error.

**Figure 3:**
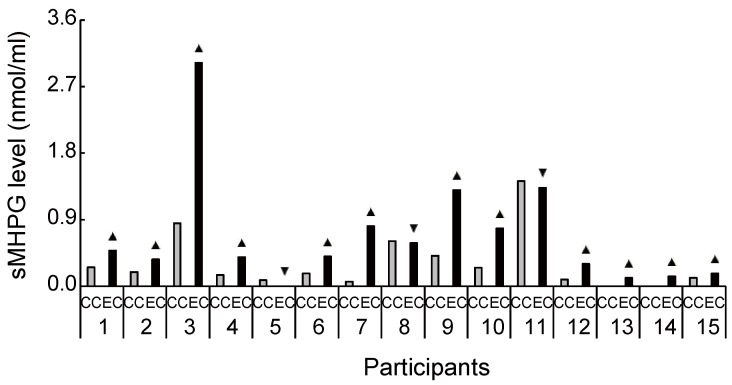
Increased and decreased ratio of salivary 3-methoxy-4-hydroxyphenylglycol level between conditions for each participant. Despite the large interindividual variance, 12 out of 15 participants had an increased ratio of 3-methoxy-4-hydroxyphenylglycol level during exercise compared with the control condition. sMHPG, salivary 3-methoxy-4-hydroxyphenylglycol; CC, control condition; EC, exercise condition; ▲, increase ratio in EC compared with CC; ▼, decrease ratio in EC compared with CC.

**Figure 4:**
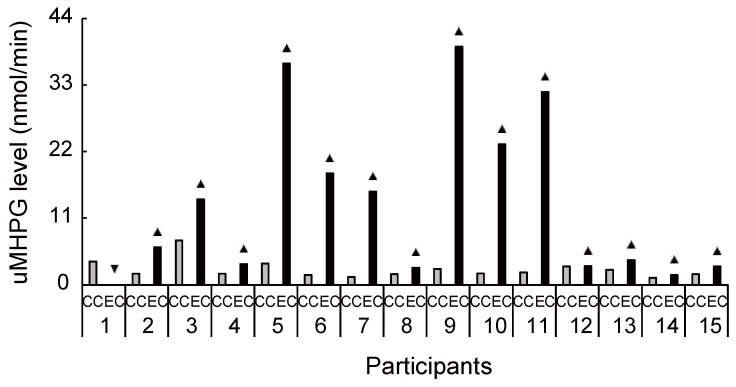
Increased and decreased ratio of urinary 3-methoxy-4-hydroxyphenylglycol level between conditions for each participant. Despite the large interindividual variance, 14 out of 15 participants had an increased ratio of 3-methoxy-4-hydroxyphenylglycol level during exercise compared with the control condition. uMHPG, urinary 3-methoxy-4-hydroxyphenylglycol; CC, control condition; EC, exercise condition; ▲, increase ratio in EC compared with CC; ▼, decrease ratio in EC compared with CC.

**Figure 5:**
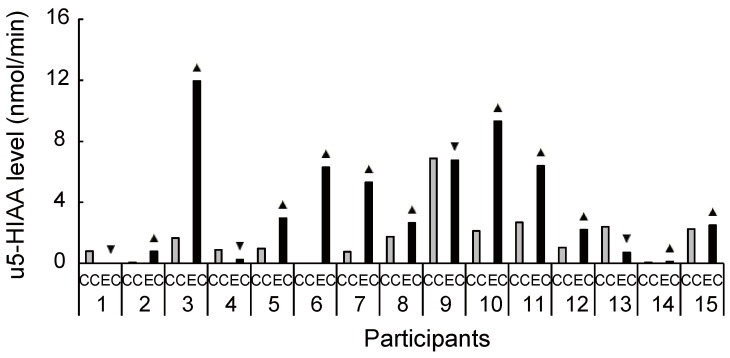
Increased and decreased ratio of urinary 5-hydroxyindoleacetic acid level between conditions for each participant. Despite the large interindividual variance, 11 out of 15 participants had an increased ratio of 5-hydroxyindoleacetic acid level during exercise compared with the control condition. u5-HIAA, urinary 5-hydroxyindoleacetic acid; CC, control condition; EC, exercise condition; ▲, increase ratio in EC compared with CC; ▼, decrease ratio in EC compared with CC.

**Figure 6:**
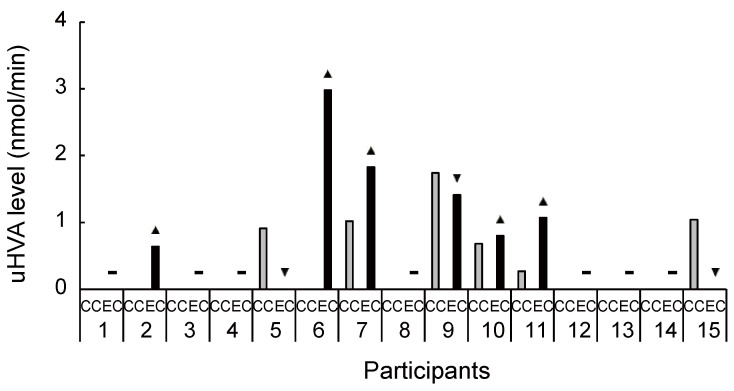
Increased and decreased ratio of urinary homovanillic acid level between conditions for each participant. Although 5 participants had an increased ratio of homovanillic acid level in urine during exercise compared with the control condition, in most participants homovanillic acid was not detected in any condition. Homovanillic acid in urine may not be used as a biomarker of dopamine in the central nervous system. uHVA, urinary homovanillic acid; CC, control condition; EC, exercise condition; ▲, increase ratio in EC compared with CC; ▼, decrease ratio in EC compared with CC; −, no change.

Biochemical HPLC analyses showed significantly higher sMHPG levels in the EC than in the CC (Figure 2A: *t*_(14)_ = 2.48, *p* = 0.027, *d* = 0.61). As Figure 3 shows, most individuals had an increased sMHPG ratio after rhythmic exercise. The condition differences in urinary monoamine are shown in Figure 2B–D. Compared with the CC, rhythmic exercise caused a significant increase in uMHPG (Figure 2B: *t*_(14)_ = 3.18, *p* = 0.007, *d* = 1.15) and u5-HIAA levels (Figure 2C: *t*_(14)_ = 2.55, *p* = 0.023, *d* = 0.79). As Figure 4 and Figure 5 show, most individuals showed an increased uMHPG and u5-HIAA ratio after rhythmic exercise. These *t*-values were higher than the adjusted significance level threshold (*t*_(14)_ =2.47) obtained in the permutation test. In contrast, there were no significant between-condition differences in uHVA levels (Figure 2D: *t*_(14)_ = 0.86, *p* = 0.406, *d* = 0.27). As Figure 6 shows, most individuals did not show differences in uHVA levels between CC and EC. These results indicate that the rhythmic exercise of long-rope jumping increased the s/uMHPG and u5-HIAA levels.

**Figure 7:**
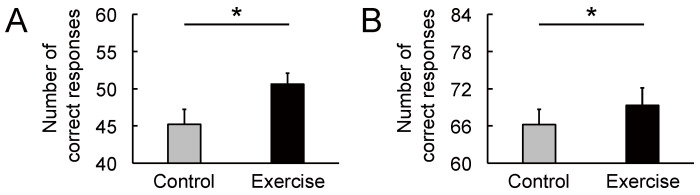
Effect of rhythmic rope jumping exercise on Stroop performance. (**A**) In the congruent word task, rhythmic exercise increased the rate of correct responses compared with the control condition. (**B**) In the incongruent word task, rhythmic exercise increased the rate of correct responses compared with the control condition. Parameters are indicated as mean (*SE*). * *p* < 0.05. *SE*, standard error.

**Figure 8:**
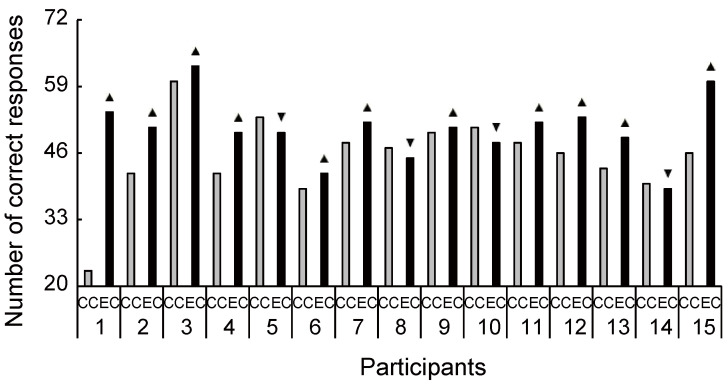
Increased and decreased ratio of Stroop congruent performance between conditions for each participant. Despite the large interindividual variance, 11 out of 15 participants had an increased ratio of Stroop performance during exercise compared with the control condition. CC, control condition; EC, exercise condition; ▲, increase ratio in EC compared with CC; ▼, decrease ratio in EC compared with CC.

**Figure 9:**
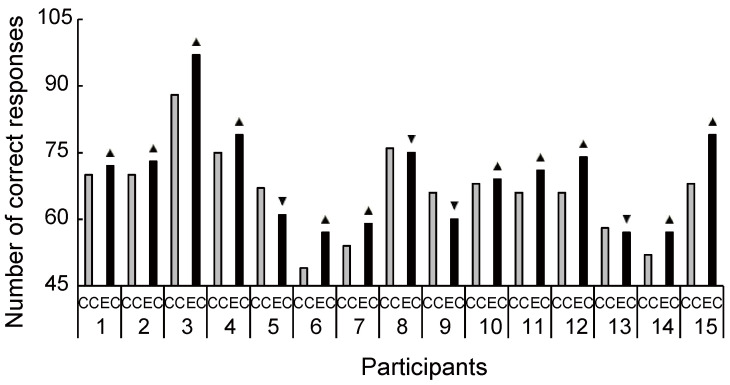
Increased and decreased ratio of Stroop incongruent performance between conditions for each participant. Despite the large interindividual variance, 11 out of 15 participants had an increased ratio of Stroop performance during exercise compared with the control condition. CC, control condition; EC, exercise condition; ▲, increase ratio in EC compared with CC; ▼, decrease ratio in EC compared with CC.

The cognitive data are shown in Figure 7. There were significant differences between the two conditions in the congruent (Figure 7A: *t*_(14)_ = 2.45, *p* = 0.028, *d* = 0.75) and incongruent word tasks (Figure 7B: *t*_(14)_ = 2.39, *p* = 0.032, *d* = 0.30). As Figure 8 and Figure 9 show, most individuals increased their Stroop performance after rhythmic exercise. These *t*-values were higher than the adjusted significant level threshold (*t*_(14)_ = 2.25) obtained in the permutation test. These results indicate that participants showed higher attention performance after the rhythmic exercise of long-rope jumping than after the CC.

We also investigated the correlation between s/uMHPG levels and u5-HIAA levels and Stroop performance (Table 2). The u5-HIAA levels were positively correlated with the s/uMHPG levels in the rhythmic EC (sMHPG: *r* = 0.80, *p* < 0.001, uMHPG: *r* = 0.58, *p* = 0.022). The absolute Pearson’s *r* values were higher than the adjusted significance level threshold (|*r*| = 0.54) obtained in the permutation test. In contrast, such a correlation with sMHPG levels was not significant in the CC (sMHPG: *r* = 0.32, *p* = 0.241, uMHPG: *r* = 0.10, *p* = 0.721). This shows that higher 5-HIAA levels are linked to higher levels of MHPG.

In subsequent correlation analyses including sex, age, and education as the control variables (Table 2), sMHPG levels were positively correlated with correct responses in the congruent word task of the Stroop test in the EC (*r* = 0.61, *p* = 0.036). The absolute Pearson’s *r* value was higher than the adjusted significance level threshold (|*r*| = 0.54) obtained in the permutation test. In contrast, such a correlation was not found for the CC (*r* = 0.26, *p* = 0.417). These results indicate that enhancement of the MHPG level 30 min after completion of the rhythmic exercise of long-rope jumping is linked to higher attention performance.

## 4. Discussion

This study aimed to investigate the association between the rhythmic exercise of long-rope jumping and central monoaminergic and cognitive functions. The main findings revealed that after the rhythmic rope jumping exercise, participants showed (1) a significant reduction of anxiety scores; (2) increased s/uMHPG and u5-HIAA levels; and (3) improved Stroop performance. Moreover, the higher release of 5-HIAA was associated with higher MHPG levels in the EC. In addition, the MHPG levels that were increased by the rhythmic rope jumping exercise were associated with higher attention performance on the Stroop task. Our results indicate that long-rope jumping may affect cognitive function by activating the norepinephrinergic and serotonergic systems and their interactions.

Consistent with previous findings [34,35], this study revealed that the rhythmic rope jumping exercise significantly reduced anxiety scores compared to the CC. The improvement in anxiety score might be explained by the decrease of hypothalamic corticotropin-releasing factor and activation of the central monoaminergic system (e.g., norepinephrine and serotonin) [36], both of which are based on the antianxiety and antidepressant effects of exercise. Psychologically, exercise is also associated with a self-efficacy improvement through progressive positive feedback, such as fitness gains [36]. The multimodal nature of exercise may contribute to improving anxiety in our study.

In contrast to previous studies using aerobic exercise showing significant improvement in depressive symptoms of high-depressive participants [37,38], we did not find similar improvement in the POMS depression score of healthy participants. One possible reason is the initial level of depression (e.g., high-depressive participants vs. healthy participants). Another possible reason is that continued rope jumping exercise may strongly reduce depression levels.

Concerning monoaminergic functions, s/uMHPG and u5-HIAA levels and their interaction were found to be increased by rhythmic rope jumping exercise. These findings can be interpreted as central norepinephrinergic and serotonergic activation due to long-rope jumping. Previous studies have reported that exhaustive exercise is associated with higher serotonin synthesis [9,39] and lower norepinephrine levels in the brain [40], indicating that monoamine changes are implicated in the central fatigue mechanism. However, one study reported that after supplementation with 2-μM L-tryptophan (serotonin precursor), nerve terminals took up the serotonin over a 60-min period, rapidly metabolizing it to 5-HIAA to return the concentration of serotonin to its original level after 90 min [41]. Another study emphasized that exercise performance is not influenced by fluoxetine (selective serotonin reuptake inhibitor) [42]. These aspects of serotonin could contribute to increased brain plasticity [43,44] but not induce central fatigue [9,41,42]. In particular, the increased u5-HIAA levels due to the rhythmic exercise of long-rope jumping may help facilitate neuroplasticity. Alternatively, the heightened u5-HIAA levels may be related to cognitive demand and motor plan in long-rope jumping. The serotonergic system projects from raphe nuclei to the precuneus and the hippocampus [45]. The precuneus is involved in attention shift and timing function [46,47,48] and plays an important role in visuospatial imagery for body movement control [49,50,51]. Malouin et al. reported activation of the precuneus in imagery tasks of walking with obstacles through a virtual environment [52], suggesting the involvement of the precuneus in efficient predictive adaptation of postural control, motor coordination, spatial orientation, and reaction to moving objects/persons. Moreover, the hippocampus has been implicated in the processing capacity of spatial information as well as attention [53,54]. In addition, neuroimaging and neurophysiological studies have showed that serotonergic modulation influences motor planning and sensory perception (e.g., rhythm and timing) [55,56]. The accumulation of training in motor skills involving high cognitive demand may strongly influence the precuneus and hippocampus associated with the serotonergic system, presumably because visuospatial processing, coordinating movements to maintain balance and rhythm, and attention shift are essential for long-rope jumping.

Interestingly, although increased dopamine availability in the brain has an exercise performance-enhancing effect [57], we could not detect uHVA excretion in most individuals, nor were there significant differences between the two conditions. Dopaminergic neurons are restricted to the nigrostriatal pathway [43], suggesting that dopamine content is much lower in other regions, except for the striatum [58]. In contrast, serotonergic and norepinephrinergic neurons are localized in the whole brain [58]. Therefore, higher u5-HIAA and uMHPG excretion would be expected as these metabolites are transported more from the whole brain via the OAT3, but not HVA excretion. Thus, uHVA excretion may not function well as the central dopamine biomarker in our study.

Further analyses revealed that higher sMHPG levels were associated with better Stroop performance in the rhythmic rope jumping exercise, indicating a rhythmic exercise-specific link between processes controlling attention and the norepinephrinergic system. This result suggests that long-rope jumping may facilitate attentional performance by central norepinephrinergic activation.

The norepinephrinergic pathway projects from the locus coeruleus to the whole brain [59]. A pharmacological study reported that after clonidine microinjection in rats, the reduction of locus coeruleus-norepinephrinergic system activity reduces prefrontal-dependent visuospatial attention performance [60], indicating a role of the norepinephrinergic system in attention associated with locus coeruleus-prefrontal circuit. In addition, aerobic exercise is associated with the activation of these regions [61,62]. Although we did not evaluate these brain functions, these aspects of the rhythmic exercise of long-rope jumping may enhance the connectivity between the locus coeruleus and the prefrontal cortex, associated with the ascending norepinephrinergic pathway. The increased peripheral MHPG levels and their association with attention performance in our study suggest that long-rope jumping may enhance central norepinephrine release for improved attention.

Finally, we provided the first evidence of a positive correlation between uMHPG levels and incongruency processing, as measured by the Stroop task in healthy participants in the CC. Since Stroop incongruent condition requires more attention and control of competitive responses [63], the higher uMHPG levels may be fast to respond to the Stroop incongruent effect. Although baseline sMHPG levels were associated with effort performance on the Uchida-Kraepelin test [22], baseline uMHPG may predict effortful attention on the Stroop interference. Therefore, baseline uMHPG could serve as a non-invasive biological marker for detecting central norepinephrinergic activity and a useful predictive marker for arousal and attention in healthy participants.

Our study has several limitations. As small sample size limits statistical power, the results in our study might be considered as preliminary. However, our study size was comparable to those in some biochemical sports trials [64,65] and, as indicated by the interindividual differences in Figure 3, Figure 4, Figure 5, Figure 6, Figure 8 and Figure 9, increased s/uMHPG and u5-HIAA levels and improved Stroop performance were observed in EC participants, suggesting that the changes in central norepinephrinergic-serotonergic systems and attention performance were caused by the rhythmic rope jumping exercise. Moreover, while the effects of long-rope jumping have been demonstrated, it is still unclear which aspect of the exercise is effective. For example, CC participants did not undergo any intervention, which limits the interpretation of our findings. That is, social interaction, as well as the rope jumping exercise, may have partially influenced the EC participants. However, it is unlikely that our exercise effects are merely due to an increase in social interaction because the two conditions did not differ in social orientation scores. Furthermore, the present study was unable to recruit a sufficient number of participants for other aerobic exercises of similar intensity (e.g., walking and dance) to further clarify the effects of rhythmic rope jumping on monoamine levels and cognitive functions. Therefore, the specific effects on aerobic demand or more complex cognitive demands of the motor planning involved in rope jumping remain unclear and should be investigated in future studies. Finally, there were few female participants in our sample. Therefore, the present results may not be generalizable to both sexes. Future research should focus on these issues to confirm or reject our findings.

## 5. Conclusions

We found that the rhythmic exercise of long-rope jumping enhances cognitive performance and central monoaminergic system function through central norepinephrinergic activation. Biochemically, the increased s/uMHPG was associated with higher u5-HIAA levels in the rhythmic rope jumping exercise. Moreover, the behavioral results indicated exercise-specific improved attention performance, suggesting that long-rope jumping may affect the central norepinephrinergic system for attention improvement. The main findings of the present study shed new light on how long-rope jumping possibly influences the central monoaminergic system.

## Figures and Tables

**Table 1:** Psychological results in both conditions.

Scale	Control	Exercise	*p*-Value
POMS anxiety	7.7 (1.2)	4.0 (0.7)	<0.001
POMS depression	3.9 (0.8)	2.9 (0.7)	0.267
POMS anger	1.8 (0.5)	2.1 (1.1)	0.832
POMS vigour	7.9 (1.1)	7.7 (0.9)	0.909
POMS fatigability	10.1 (0.8)	11.5 (1.1)	0.257
POMS confusion	6.2 (0.7)	5.0 (0.8)	0.132
Individual orientation	23.9 (0.7)	25.1 (0.8)	0.098
Social orientation	33.7 (1.2)	34.9 (1.5)	0.342

Parameters are indicated as the mean (*SE*). *p*-values are from *t*-tests on condition differences. POMS, profile of mood states; *SE*, standard error.

**Table 2:** Correlations between monoamine metabolites and attention.

Pair of Variables	Control	Exercise
(A) Correlation with u5-HIAA		
sMHPG	0.32	0.80 ***
uMHPG	0.10	0.58 *
(B) Correlation with Stroop performance		
Congruent word task		
sMHPG	0.26	0.61 *
uMHPG	0.44	0.03
u5-HIAA	0.35	0.38
Incongruent word task		
sMHPG	0.27	0.50
uMHPG	0.59 *	−0.30
u5-HIAA	−0.25	0.38

* *p* < 0.05, *** *p* < 0.001. u5-HIAA, urinary 5-hydroxyindloeacetic acid; sMHPG, salivary 3-methoxy-4-hydroxyphenylglycol; uMHPG, urinary 3-methoxy-4-hydroxyphenylglycol.

## Data Availability

The raw data that support the findings of this study are available from the corresponding author upon reasonable request.
